# The effect of COVID-19 on the academic performance of Zayed University students in the United Arab Emirates

**DOI:** 10.3389/fpsyg.2023.1199684

**Published:** 2023-09-06

**Authors:** Sharifa AlBlooshi, Linda Smail, Alyaa Albedwawi, Mariam Al Wahedi, Maha AlSafi

**Affiliations:** ^1^Department of Health Sciences, College of Natural and Health Sciences, Zayed University, Dubai, United Arab Emirates; ^2^Computational Systems Program, College of Interdisciplinary Studies, Zayed University, Dubai, United Arab Emirates; ^3^Department of Arabic, Islamic and Legal Studies, College of Humanities and Social Sciences, Zayed University, Abu Dhabi, United Arab Emirates; ^4^Department of Community Health, Abu Dhabi Public Health Center, Abu Dhabi, United Arab Emirates; ^5^Department of Public Health Research, Abu Dhabi Public Health Center, Abu Dhabi, United Arab Emirates

**Keywords:** COVID-19, academic performance, UAE students, level of satisfaction, online teaching

## Abstract

**Purpose:**

The outbreak of the coronavirus (COVID-19) pandemic led to significant changes across various sectors, including the field of education. In response to the pandemic, educational institutions worldwide, including Zayed University in the United Arab Emirates (UAE), transitioned to online learning. This study aimed to investigate the impact of COVID-19 on the academic performance of students in the UAE and their satisfaction with remote learning, while also examining gender differences in these variables.

**Methods:**

This study used a quantitative research design in which a questionnaire was used to collect data. The study employed a snowball sampling method to recruit a total of 1,780 male and female students aged 18 and above from Zayed University in the UAE. The collected data were analyzed using appropriate statistical techniques.

**Results:**

This study revealed that students at Zayed University maintained a good level of academic performance (*M* = 3.34, SD = 0.76) during the COVID-19 pandemic and were satisfied with online learning (*M* = 3.48, SD = 0.84) during the COVID-19 pandemic. A significant positive correlation was observed between students’ academic performance and their level of satisfaction with online teaching during the COVID-19 pandemic (*p* < 0.001). Furthermore, no significant differences were found between gender and both academic performance and level of satisfaction with online learning. Finally, we found that more males leaned toward online learning while more females leaned toward face-to-face learning.

**Conclusion:**

This study contributes to understanding the impact of COVID-19 on students’ academic performance and satisfaction with remote learning in the UAE context. The findings highlight the significance of student satisfaction for successful online learning and emphasize the need for adequate resources and the maintenance of education quality.

## Introduction

The rapid and unexpected outbreak of COVID-19 dramatically altered daily life across the globe. Every sector faced unprecedented disruption, and education was no exception. Within a short span, traditional in-person classrooms were rendered untenable, prompting a swift transition to digital platforms for learning and teaching.

The United Arab Emirates (UAE), known for its fast-paced development and technological advancements, was quick to adapt to these changes. Institutions across the country, including Zayed University, moved to online learning as a preventive measure against the spread of the virus (Liaqat, 2021). This move not only safeguarded the health of the educational community but also ensured the continuity of education for students.

This sudden transition, however, brought forth several challenges. It underscored the need to evaluate the resilience and adaptability of our education systems in the face of such emergencies (Crawford and Cifuentes-Faura, 2022). Moreover, it raised questions about the quality and efficacy of online learning and the disparities in access and performance that may arise from it.

While there is a substantial body of research globally on online learning, its benefits, and associated challenges, the COVID-19 pandemic presented a unique context. The swift, unplanned, and comprehensive shift to online learning necessitated by a global emergency added new dimensions to the discussion. In particular, the role of online learning in influencing students’ attitudes toward larger societal issues, notably sustainability, emerged as an important area of exploration following the interdisciplinary approach encouraged by Lafuente-Lechuga et al. (2023) and Cifuentes-Faura and Noguera-Méndez (2023).

Specifically, there is a scarcity of studies focusing on the impact of this sudden shift on education in the UAE. In particular, there is a need to explore variations in academic performance and satisfaction across different demographic groups in this setting, especially gender. This gap in the literature is significant considering the cultural, social, and economic contexts of the UAE and their potential impact on learning outcomes.

The sudden shift to online learning required a close look at its impact on students, in particular on their academic performance and satisfaction. The specifics of these effects could vary depending on the demographic characteristics of students, their field of study, and the resources available to them. The examination of such variables in the context of the UAE forms the essence of this study.

The present study aims to address this gap. It seeks to present a unique perspective from a rapidly developing nation that swiftly transitioned into online learning due to the pandemic. By examining the effects of the pandemic-induced transition to online learning on academic performance and satisfaction, with a specific focus on gender variations, this study hopes to add to the global understanding of the impacts of the COVID-19 pandemic on education.

The primary objective of this study is to investigate the impact of the COVID-19 pandemic on the academic performance and level of satisfaction with remote learning among students in governmental universities in the United Arab Emirates. Furthermore, we aim to examine the differences in these variables across gender groups.

## Literature review

The relationship between online education, student satisfaction, and academic performance has been the subject of numerous studies (Uusiautti et al., 2017; Harsasi and Sutawijaya, 2018; Han et al., 2021; She et al., 2021; Yu, 2022). However, the unplanned shift to online learning during the COVID-19 pandemic introduced new dynamics into this relationship (del Arco et al., 2021; Ramos-Pla et al., 2022).

Online learning offers several benefits, including increased flexibility and reduced costs for institutions (Wang, 2015; Liaqat, 2021). However, the transition to this model also presents challenges, such as technical difficulties and accessibility issues (Leszczyński et al., 2018; Liaqat, 2021). This shift was facilitated by educational software like Blackboard LMS and Microsoft Teams, used for online exams and assessments. To maintain the integrity of assessments, institutions implemented proctoring tools like Lockdown Browser and Respondus Monitor, presenting a learning curve for novices (Adedoyin and Soykan, 2020; Gawanmeh et al., 2021). Beyond academic performance and satisfaction, the role of online learning in shaping attitudes toward larger societal issues, such as sustainability, is an increasingly relevant area of study. Lafuente-Lechuga et al. (2023) emphasize the importance of integrating Sustainable Development Goals in the curriculum to foster a sustainable world. They advocate for a cross-disciplinary approach to enable students to analyze sustainability issues using different academic perspectives. Likewise, Cifuentes-Faura and Noguera-Méndez’s (2023) investigation into the attitudes of Economics and Business students toward sustainability underscores this relevance. They found that while students are aware of environmental problems, there is a lack of consensus on the necessary actions to overcome them. They also revealed a gender divide, with female students showing more favorability toward sustainability than their male counterparts.

These challenges were amplified for students from socio-economically disadvantaged backgrounds who might struggle to meet the technological requirements of online learning and deal with distractions in the home environment (Adedoyin and Soykan, 2020; Novikov, 2020; UAE Government Portal, 2020). Subjects that require hands-on learning, such as medical sciences, arts, and engineering, also face unique hurdles in an online format (Iqbal et al., 2015; Leszczyński et al., 2018; Daniel, 2020).

Educators grappled with issues too, such as ensuring fair grading during the pandemic, transitioning to effective remote teaching, and maintaining student engagement (Adedoyin and Soykan, 2020; D’Oliveira et al., 2022; Galli et al., 2022; AlBlooshi et al., 2023). This transitional phase also required appropriate faculty training to utilize digital tools and online course designs effectively, which was a crucial factor in the teaching adaptation process during the pandemic (del Arco et al., 2021; Ramos-Pla et al., 2021). Furthermore, the success of online teaching is not solely dependent on the aforementioned factors, but also on the adaptability and preparedness of the educators, which might significantly influence students’ academic performance and satisfaction levels (Ramos-Pla et al., 2021, 2022).”

Students’ satisfaction with online learning is influenced by multiple factors, such as student-teacher interactions, peer interactions, quality of assessments, student self-efficacy, and individual learning abilities (Uusiautti et al., 2017; Harsasi and Sutawijaya, 2018; Han et al., 2021; She et al., 2021; Yu, 2022).

In the UAE, the abrupt shift to online learning due to the pandemic received mixed responses. Some studies found high student satisfaction (Almusharraf and Khahro, 2020; Chen et al., 2020; Sharma et al., 2020; Gawanmeh et al., 2021; Liaqat, 2021), while others reported a preference for traditional face-to-face classes or blended learning (Almuraqab, 2020; Hussein et al., 2020). Moreover, students admitted that the unavailability of proper tools for remote learning at home obstructed their educational experience, particularly for hands-on subjects (Dinh and Nguyen, 2020; Gawanmeh et al., 2021).

However, these studies largely overlooked gender-based differences in experiences and outcomes, a gap this study seeks to address. By focusing on this unexplored area, our research aims to contribute to the existing literature by providing an in-depth examination of the impact of COVID-19 on student academic performance and satisfaction in the UAE, specifically at Zayed University. The unique dimension our study introduces is the examination of these variables across gender, a facet receiving limited attention in previous research. By doing so, our study not only offers a fresh lens on understanding the implications of pandemic-induced online education but also illuminates the gender-specific variations in academic performance and satisfaction, offering potentially valuable insights for educational institutions in similar settings.

## Methodology and data

This study used a quantitative research design employing a digital questionnaire to investigate the effects of the COVID-19 pandemic on the academic performance of Zayed University students and their level of satisfaction with online classes. An online questionnaire is the best way to reach a larger sample to collect data on their overall average experience. The anonymity of the questionnaire also allows the students to be more honest in giving their opinion.

## Participants and sampling

This study was carried out in the UAE from February 2022 to April 2022. It included 1,780 participants from Zayed University, of which 183 were male and 1,597 were female. The participants were all above 18 years old. Snowball sampling was used to collect the sample due to its convivence and swiftness. The questionnaire was created using Google Forms and translated into Arabic. The principal investigator sent the questionnaire (in both languages) via ZU email to students and ZU faculties, who then shared it with their peers via email and social media. A total of 1,805 questionnaires were completed by ZU students. Questionnaires with missing answers were removed leaving 1,780 fully completed questionnaires.

### Data collection instruments

Our research employed a questionnaire adapted from Hashemi (2021), which drew from earlier work (Almazova et al., 2020; Almusharraf and Khahro, 2020; Elhadary et al., 2020). The questionnaire was provided in two languages, English and Arabic, to facilitate diverse participant responses. The questionnaire was made up of 45 items, divided into four thematic sections.

The first section (7 items) gathered sociodemographic information about the participants, including details such as age, gender, field of study, and year of study. The second section, featuring a single item, focused on identifying participants’ preferred mode of instruction – online, face-to-face, or blended learning.

In the third section (17 items), we explored the impact of COVID-19 on the academic performance of the participants. Questions delved into areas such as changes in grade point average (GPA), study habits, concentration levels, and any perceived changes in understanding and retention of course material during online learning.

The fourth section (12 items) assessed participants’ satisfaction levels with online learning during the pandemic. Items in this section were designed to assess aspects such as ease of use of online platforms, interaction with faculty and peers, the effectiveness of online assessments, and the overall quality of the online learning experience.

The final section (8 items) consisted of general questions related to online teaching, including factors like the effect on student-teacher communication, the suitability of the physical working environment for online learning, changes in studying hours, and shifts in student responsibility in the online learning context.

Our questionnaire initially underwent a thorough review by subject-matter experts. Following this, it was translated into Arabic and subsequently back-translated into English. Both versions were then examined once again by the experts, and their invaluable feedback was carefully incorporated into the final questionnaire.

We then validated our questionnaire through a pilot test involving a sample of 30 students. The pilot test allowed us to ensure the clarity and appropriateness of the questionnaire items. The Cronbach’s alpha was 0.940 indicating acceptable internal consistency.

### Data analysis

The data were analyzed using IBM SPSS Statistics 27 (IBM, Armonk, NY, USA). Frequencies, percentages, mean scores, and standard deviations were determined using descriptive statistics. Inferential statistical analysis was utilized to investigate the existence of significant differences among variables. We examined the academic performance of students and their level of satisfaction with online learning across genders.

### Ethical clearance

This study was approved by the Research Ethics Committee (REC), Office of Research at Zayed University Ethics Application Protocol Code ZU21_169_F, and the Ministry of Health and Prevention Research Ethics Committee, Ethics Application Protocol Code MOHAP/DXB-REC/ JJF/No. 05/2022. All participants joined the study voluntarily after signing an informed consent form. The form described the goals and objectives of the study and what was expected of the participants and asked for their consent to the use and publishing of the data. Participants’ confidentiality and anonymity were maintained, and they were not subjected to any harm.

## Results

The participant demographics, as outlined in Table 1, reveal a considerable majority of female participants at 89.7% (1,597 out of 1,781 participants), with males representing 10% (183 participants). Over half of the participants were from Dubai (58.7%) followed by Abu Dhabi (15.3%), and then Sharjah (12.9%). Most participants resided in urban areas (87.5%). The majority were singles (84.1%). The majority, 95.9%, of the participants were UAE nationals while only 4.1% were non-UAE nationals. The participants came from different colleges. The most prevalent college is the College of Natural Health and Sciences (28.9%) followed by the College of Business (20%), then the College of Technological Innovation (14.6%).

**Table 1:** Demographic characteristics.

		Frequency	Percent	Valid percent	Cumulative percent
Gender	Female	1,598	89.7	89.7	89.7
	Male	183	10.3	10.3	100.0
	Total	1,781	100.0	100.0	
Emirate	Abu Dhabi	272	15.3	15.3	15.3
	Dubai	1,045	58.7	58.7	73.9
	Sharjah	229	12.9	12.9	86.8
	Ras Al Khaimah	47	2.6	2.6	89.4
	Ajman	117	6.6	6.6	96.0
	Umm Al Quwain	49	2.8	2.8	98.8
	Fujairah	22	1.2	1.2	100.0
Nationality	UAE National	1,708	95.9	95.9	95.9
	Non-UAE National	73	4.1	4.1	100.0
	Total	1,781	100.0	100.0	
Residential area	Urban	1,559	87.5	87.5	87.5
	Rural	222	12.5	12.5	100.0
	Total	1,781	100.0	100.0	
Marital status	Married	242	13.6	13.6	13.6
	Single	1,498	84.1	84.1	97.7
	Divorced	9	0.5	0.5	98.2
	Widowed	16	0.9	0.9	99.1
	Separated	16	0.9	0.9	100.0
Your college	College of Arts & Creative Enterprises	184	10.3	10.3	10.3
	College of Business	356	20.0	20.0	30.3
	College of Communication & Media Sciences	158	8.9	8.9	39.2
	College of Humanities & Social Sciences	206	11.6	11.6	50.8
	College of Natural & Health Sciences	509	28.6	28.6	79.3
	College of Education	108	6.1	6.1	85.4
	College of Technological Innovation	260	14.6	14.6	100.0
	Total	1,781	100.0	100.0	

Table 2 encapsulates preferences for teaching methods, segmented by gender and age. Among the 8.1% who are aged 18–25, more male participants (4.7%) preferred online learning while the other 3.5% of male respondents preferred face-to-face classes. In the same age group, out of 81.9%, more females (50.7%) preferred face-to-face classes while 31.2% of female participants preferred online learning. In the age range of 25–30, among the 0.7% of males, more males (0.5%) preferred online teaching again, compared to the 0.2% that preferred face-to-face. 2.4% of the female participants of the same age range preferred face-to-face compared to the 1.5% of females that preferred online teaching out of a total of 3.9%. For those above 30 years old, among 1.5, 0.8% of male participants preferred online teaching while 0.6% preferred face-to-face. 1.2% of female participants who are above 30 years old preferred online teaching and 2.7% of female participants favored face-to-face teaching out of the total 3.9%. Ultimately, out of the total 10.3% of males, more males leaned toward online learning (6.0%) than face-to-face (4.3%). While out of the total 89.7% more females leaned toward face-to-face learning (55.8%) than online (33.9%). Overall, most of the total sample favored face-to-face teaching (60.1%) while the remaining 39.9% of participants preferred remote learning.

**Table 2:** Descriptive statistics of preference of teaching mode by gender and age.

Age			Female	Male	Totals
18–25	Preference	Online teaching	31.2%	4.7%	35.9%
		Face-to-face teaching	50.7%	3.5%	54.2%
	Total		81.9%	8.1%	90.1%
25–30	Preference	Online teaching	1.5%	0.5%	1.9%
		Face-to-face teaching	2.4%	0.2%	2.6%
	Total		3.9%	0.7%	4.5%
Above 30	Preference	Online teaching	1.2%	0.8%	2.1%
		Face-to-face teaching	2.7%	0.6%	3.3%
	Total		3.9%	1.5%	5.4%
Total	Preference	Online teaching	33.9%	6.0%	39.9%
		Face-to-face teaching	55.8%	4.3%	60.1%
	Total		89.7%	10.3%	100%

Participants were asked to rate items regarding the effects of COVID-19 on their academic performance during the pandemic on a Likert scale ranging from 1 for strongly disagree to 5 for strongly agree. The mean score of each item is given in Table 3. The scores ranged between 3.09 and 3.68, indicating that most participants either strongly agreed or agreed on each item about academic performance. While the highest score was related to item 14:” during the COVID-19 pandemic, I completed the online study assignments assigned by the teacher on time.,” the lowest score is associated with item 17: “I have experienced difficulty communicating with my teachers during the COVID-19 crisis…” While there was no issue with completing required assignments online, the participants experienced issues communicating with their faculty.

**Table 3:** The effects of COVID-19 on the academic performance of the students.

No	Items	Strongly disagree N%	Disagree N%	Neutral N%	Agree N%	Strongly agree N%	Mean	S.D
1	I enjoyed completing online courses.	212 11.9	224 12.6	615 34.6	440 24.7	289 16.2	3.21	1.21
2	Performing well in online courses made me feel good about myself.	164 9.2	173 9.7	543 30.5	556 31.2	344 19.3	3.42	1.17
3	I felt that online education was a good chance to enhance my academic performance.	189 10.6	258 14.5	507 28.5	486 27.3	340 19.1	3.30	1.23
4	Completing online courses moved me closer to attaining my career goals.	175 9.8	268 15.1	577 32.4	467 26.2	293 16.5	3.24	1.19
5	I felt able to perform well in online courses.	173 9.7	264 14.8	492 27.6	530 29.8	321 18.0	3.32	1.21
6	During the COVID-19 outbreak, I did all my activities successfully online.	112 6.3	182 10.2	482 27.1	630 35.4	374 21.0	3.55	1.12
7	During the COVID-19 outbreak, I organized my time to do everything the teachers asked me to do.	113 6.3	186 10.4	464 26.1	651 36.6	366 20.6	3.55	1.12
8	During the COVID-19 outbreak, my academic performance improved.	168 9.4	247 13.9	544 30.6	510 28.7	311 17.5	3.31	1.19
9	during the COVID-19 outbreak, I have acquired more knowledge by taking online classes.	146 8.2	270 15.2	530 29.8	550 30.9	284 16.0	3.31	1.15
10	During the COVID-19 outbreak, I have improved my communication skills by taking classes online.	174 9.8	267 15.0	499 28.0	557 31.3	283 15.9	3.29	1.19
11	During the COVID-19 outbreak, I improved my creativity skills.	128 7.2	236 13.3	561 31.5	574 32.2	281 15.8	3.36	1.12
12	During the COVID-19 pandemic, the existing functions of the online teaching platforms can meet the learning needs.	115 6.5	187 10.5	561 31.5	613 34.4	304 17.1	3.45	1.09
13	During the COVID-19 pandemic, I actively answered the teacher’s questions and participated in classroom learning.	115 6.5	198 11.1	516 29.0	634 35.6	317 17.8	3.47	1.10
14	during the COVID-19 pandemic, I completed the online study assignments assigned by the teacher on time.	90 5.1	125 7.0	476 26.7	666 37.4	423 23.8	3.68	1.07
15	I hold an entirely negative attitude toward the online platform because of some dissatisfaction with the use of the platform (such as registration trouble, slow login, etc.)	187 10.5	299 16.8	553 31.1	507 28.5	234 13.1	3.17	1.17
16	Fear and anxiety during the coronavirus lockdown affected my study plan.	167 9.4	369 20.7	541 30.4	463 26.0	240 13.5	3.13	1.17
17	I have experienced difficulty communicating with my teachers during the COVID-19 crisis.	153 8.6	400 22.5	578 32.5	424 23.8	225 12.6	3.09	1.14

The students’ satisfaction with online teaching during the COVID-19 outbreak on a Likert scale ranging from 1 for strongly disagree to 5 for strongly agree is shown in Table 4, the mean scores for the level of satisfaction ranged between 3.39 to 3.56 indicating that students mostly agreed or strongly agreed regarding the satisfaction statements. The highest score was associated with item 4 “Online courses allowed me to access a wide range of resources,” while the lowest score was associated with item 8 “I was satisfied with the online classes as they helped me achieve the course learning outcomes.” As the minimum was higher than 3 this indicates a high level of satisfaction of the participants regarding online teaching during COVID-19.

**Table 4:** Students level of satisfaction with online teaching during the COVID-19 outbreak.

No	Items	Strongly disagree	Disagree	Neutral	Agree	Strongly agree	Mean	S.D
1	I was satisfied with the webinars, seminars, and courses offered based online.	110 5.6	158 8.9	618 34.7	609 34.2	295 16.6	3.47	1.05
2	I was satisfied with the instructors’ follow-up in each session of online teaching.	101 5.7	165 9.3	578 32.5	659 37.0	277 15.6	3.48	1.04
3	I was satisfied with the instructors’ various online teaching approaches.	98 5.5	162 9.1	560 31.5	676 38.0	284 16.0	3.50	1.04
4	I was satisfied with the online classes as they helped me achieve the course learning outcomes.	129 7.2	202 11.3	576 32.4	598 33.6	275 15.4	3.39	1.10
5	I was pleased with the quality of teachers’ work in online courses.	100 5.6	155 8.7	540 30.3	684 38.4	301 16.9	3.52	1.05
6	I was satisfied with teachers’ motivation in online courses.	104 5.8	166 9.3	548 30.8	653 36.7	309 17.4	3.50	1.07
7	I was satisfied with the convenience of the online learning environment.	117 6.6	176 9.9	539 30.3	623 35.0	325 18.3	3.48	1.10
8	Online courses allowed me to access a wide range of resources.	89 5.0	152 8.5	543 30.5	673 37.8	323 18.1	3.56	1.04
9	I was satisfied with fair compensation or incentives for teaching online.	110 6.2	164 9.2	635 35.7	598 33.6	273 15.3	3.43	1.05
10	I worked well together with my teachers and classmates in online courses.	93 5.2	157 8.8	541 30.4	665 37.4	324 18.2	3.54	1.05
11	I was satisfied with the content quality of my online courses.	111 6.2	178 10.0	547 30.7	642 36.1	302 17.0	3.48	1.08
12	Professional development or training for online learning made me experience a higher level of satisfaction with online teaching.	105 5.9	169 9.5	602 33.8	595 33.4	309 17.4	3.47	1.07

Table 5 shows the mean score for academic performance and students’ satisfaction level according to gender. The mean score of (*M* = 3.37, SD = 0.87) for the male participants in academic performance is slightly higher than the mean score of (*M* = 3.34, 0.75) for the female participants. Moreover, the mean score for the male participants’ satisfaction level is (*M* = 3.47, SD = 0.95). While the mean score for female participants in the satisfaction level is slightly higher (*M* = 3.49, SD = 0.83).

**Table 5:** The mean score of the academic performance and the satisfaction level by gender.

Academic performance				
Gender	*N*	Mean	Std. deviation	Std. error of mean
Male	183	3.37	0.87	0.065
Female	1,597	3.34	0.75	0.019
Total	1,780			

Satisfaction level				
Gender	*N*	Mean	Std. deviation	Std. error of mean
Male	183	3.47	0.95	0.071
Female	1,597	3.49	0.83	0.021
Total	1,780			

Table 6 lists the results of an independent samples test used to investigate the existence of a significant difference in academic performance and the level of satisfaction with online teaching of students across gender. The value of *p* for students’ academic performance was higher than the alpha value (*p* = 0.618). This reveals that there is no significant difference in the academic performance of students across gender during the COVID-19 pandemic. Moreover, the value of *p* for the level of satisfaction was higher than the alpha value (*p* = 0.876). Therefore, there is no significant difference in the students’ level of satisfaction with online teaching across gender during the COVID-19 pandemic.

**Table 6:** Independent samples test of academic performance and level of satisfaction across gender.

		*F*	Sig.	t	df	Sig. (2-tailed)	Mean difference	Std. Error difference	95% confidence interval of the difference	
									Lower	Upper
Academic performance	Equal variances assumed	3.541	0.060	−0.499	1,778	0.618	−0.02962	0.05939	−0.14611	0.08686
	Equal variances not assumed			−0.441	213.591	0.660	−0.02962	0.06725	−0.16218	0.10293
Level of Satisfaction	Equal variances assumed	4.514	0.034	0.156	1,778	0.876	0.01024	0.06575	−0.11872	0.13920
	Equal variances not assumed			0.139	214.622	0.889	0.01024	0.07354	−0.13472	0.15520

A Pearson correlation analysis was used to test the presence of a relationship between students’ academic performance and their satisfaction with online learning. As seen in Table 7, the value of *p* is less than the alpha value (*p* = 0.000 < 0.05). Therefore, a significant positive correlation exists between students’ academic performance and their satisfaction with online teaching during the COVID-19 pandemic.

**Table 7:** Correlation between academic performance and students’ level of satisfaction.

		Level of satisfaction
Academic performance	Pearson correlation	0.801**
	Sig. (2-tailed)	0.000
	*N*	1,780

**Correlation is significant at the 0.01 level (2-tailed).

Finally, Table 8 displays the frequency of ‘yes’ or ‘no’ answers regarding questions about online learning. When asked whether they find online meetings to be as effective as face-to-face meetings, 55% of students answered ‘yes’, while the other 45% answered ‘no’. Similarly, 56% agreed that online communication with their colleagues and supervisors was easier than on campus. The majority (74%) said that they receive enough training and IT support when they are online. 66% found attending conferences and scientific meetings virtually effective. 60.1% preferred studying on campus over online classes. 69% have their own physical working environment in their home. 57% thought that they needed extra hours of studying in an online setting. Finally, most of the participants (79%) thought that their responsibility to their studies was higher during online learning.

**Table 8:** Individual questionnaire answers.

Questions	Answers	Frequency	Percent	Valid percent	Cumulative percent
Did you find online meetings to be as effective as face-to-face meetings?	Yes	983	55.2	55.2	55.2
	No	798	44.8	44.8	100.0
Did you find communicating with your colleagues and supervisors was easier online than on campus?	Yes	1,001	56.2	56.2	56.2
	No	780	43.8	43.8	100.0
Did you receive enough training and IT support when you were online?	Yes	1,312	73.7	73.7	73.7
	No	469	26.3	26.3	100.0
Did you find attending conferences and scientific meetings virtually to be effective?	Yes	1,169	65.6	65.6	65.6
	No	612	34.4	34.4	100.0
Do you prefer studying online rather than studying on campus?	Yes	711	39.9	39.9	39.9
	No	1,070	60.1	60.1	100.0
Did you have your own physical working environment in your home?	Yes	1,234	69.3	69.3	69.3
	No	547	30.7	30.7	100.0
Do you think you need extra hours of studying in an online setting?	Yes	1,018	57.2	57.2	57.2
	No	763	42.8	42.8	100.0
Do you think your responsibility to your studies during the online setting was higher?	Yes	1,400	78.6	78.6	78.6
	No	381	21.4	21.4	100.0

## Discussion

Our findings revealed that regardless of age, a higher percentage of males (6.0%) leaned toward online learning compared to face-to-face learning (4.3%). Conversely, a larger proportion of females (55.8%) preferred face-to-face learning over online learning (33.9%). This aligns with a study conducted by Yu (2021) where more than 80% of females reported a preference for consistent learning methods and a dislike for online learning. On the other hand, over 85% of males preferred online learning due to its convenience. Examining the sample’s overall preferences, only 39.9% of the participants favored online classes, while the majority (60.1%) preferred face-to-face classes. This agrees with multiple studies where students leaned more toward face-to-face learning (Almuraqab, 2020; Dinh and Nguyen, 2020; Fidalgo et al., 2020; Hussein et al., 2020). It is important to note that these results may be influenced by the fact that the majority of the sample consists of female students.

Regarding academic performance during the COVID-19 pandemic, the participants achieved an overall mean score of 3.34 with a standard deviation of 0.76, indicating a good level of performance. Analyzing the data b gender, male participants obtained a slightly higher mean score of 3.37 compared to female participants with a mean score of 3.34. However, inferential statistical analysis revealed no significant difference in the academic performance of students across gender during the COVID-19 pandemic (*p* = 0.618). Similar studies also found no significant relationship between gender and academic performance in an online learning setting during the COVID-19 outbreak (Yu, 2021). It appears that students were able to maintain a good academic performance level during this sudden change regardless of gender.

Nonetheless, the transition to online learning was not without its challenges. Forty-five percent of participants reported that they perceived online classes as less effective compared to face-to-face classes, and 57% indicated that they required additional hours of studying in the online setting. Moreover, a significant majority of the participants (79%) felt a higher sense of responsibility toward their studies during online learning. These findings emphasize the need for additional student support during emergency situations like the COVID-19 pandemic. Similar studies reported that the rapid changes that came with the COVID-19 pandemic caused similar issues for students (Cifuentes-Faura et al., 2021; Faura-Martínez et al., 2022). Furthermore, these findings validate concerns raised about teachers’ preparedness to conduct remote classes (Adedoyin and Soykan, 2020; Cutri et al., 2020; Liaqat, 2021). They suggest that the quality of the education delivered online during the pandemic may not have been as satisfactory as it was prior to the pandemic.

In terms of students’ satisfaction with online learning during the COVID-19 pandemic, the mean scores for the level of satisfaction ranged between 3.39 to 3.56 indicating that students were mostly satisfied. The mean satisfaction score for the male respondents was 3.47 with a standard deviation of 0.95, while for female respondents, it was slightly higher at 3.49 with a standard deviation of 0.83. Inferential statistical analysis found no significant difference in the students’ level of satisfaction with online learning across gender during the COVID-19 pandemic (*p* = 0.876). Other studies also found no relationship (Hettiarachchi et al., 2021; Malkawi et al., 2021; Yu, 2021). A study by Malkawi et al. (2021) reasoned that this may be due to the equal provision of resources to all students and the existence of the required tools and infrastructure. However, some studies have found conflicting results regarding the relationship between gender and satisfaction with online learning, with varying conclusions on which gender exhibits higher levels of satisfaction (Aristovnik et al., 2020; Bayrak et al., 2020; Sharma et al., 2020; Yu, 2022).

Regarding the relationship between students’ satisfaction with online teaching and their academic performance, the study found that the overall mean score for academic performance was 3.34 with a standard deviation of 0.76. In contrast, participants expressed a higher level of satisfaction with online teaching, as indicated by their overall mean score of 3.48 and standard deviation of 0.84. A Pearson correlation analysis validated that there is a significant positive correlation between students’ academic performance and their level of satisfaction with online teaching during the COVID-19 pandemic (*p* < 0.001). This finding is consistent with previous studies conducted by Malkawi et al. (2021), Younas et al. (2022), and Gopal et al. (2021), which also reported a positive association between satisfaction with online teaching and academic outcomes. These findings underscore the importance of maintaining student satisfaction during online learning as it positively contributes to overall educational outcomes.

While our findings align with previous studies conducted by Yu (2021) and Fidalgo et al. (2020), and others in observing a gender-based preference in learning modes and a correlation between satisfaction and academic performance, some contrasts also emerged. For instance, our finding that the majority of students perceived online classes as less effective compared to face-to-face instruction differs from studies such as those conducted by Muthuprasad et al. (2021) which reported higher efficacy for online learning. This divergence may be attributed to the different contexts and unique challenges posed by the COVID-19 pandemic. It is also important to note that our study contributes unique insights by examining students’ experiences during the exceptional circumstances of the COVID-19 pandemic.

### Limitations

There are some limitations to these findings. Firstly, the cross-sectional design cannot investigate long-term outcomes. It also cannot explain causal relationships. However, it is useful to study multiple outcomes and to determine the need for further, long-term study. Another limitation is the risk of self-reporting bias. Participants may give out answers that they deem more socially acceptable. This also includes the risk of recall bias. Thirdly, using an online closed-ended questionnaire runs the risk that participants may not fully understand the questions or find an answer that encompasses their opinion fully and accurately. This was minimized by conducting a pilot test on a sample of 30 students to ensure the clarity of the questionnaire. Finally, the usage of the snowball sampling method makes the results not generalizable to the overall population.

## Conclusions and recommendations

Overall, our study reveals several important findings regarding online learning during the COVID-19 pandemic. First, we found that students expressed a high level of satisfaction with online learning despite the challenging circumstances. This suggests that despite the perceived decline in the quality of education, students were able to adapt and achieve positive outcomes in their academic performance.

Furthermore, our analysis demonstrated a significant positive correlation between students’ academic performance and their satisfaction with online teaching during the COVID-19 pandemic. This indicates that student satisfaction played a crucial role in their educational achievements. It emphasizes the importance of prioritizing student satisfaction in online learning environments to foster favorable educational outcomes.

Moreover, our study found no significant difference between gender and both academic performance and level of satisfaction with online learning during the COVID-19 outbreak. This suggests that both male and female students had equal access to resources and support, enabling them to achieve similar levels of academic performance and satisfaction with online learning. Future research could delve deeper into the reasons behind the observed gender preferences for online and face-to-face learning within this specific population.

Building upon these insights, our study also unveils crucial implications for gender-specific strategies in online education. We discovered that male students demonstrated a higher preference for online learning, whereas females leaned toward traditional face-to-face learning. This underlines the importance of tailored strategies in delivering online education to cater to these distinct preferences, enhancing both academic outcomes and satisfaction levels for all students.

Additionally, a significant portion of students perceived online classes as less effective than in-person ones. This calls for a deeper focus on educator training, ensuring they are equipped with the necessary pedagogical skills for online instruction. Concurrently, developing resources that guide students in navigating the online learning environment could bolster their academic experiences and outcomes.

Moreover, the increased sense of responsibility and need for additional study hours reported by the majority of students during online learning underscores the urgency for amplified student support services. Extending these services, like longer library hours or online tutoring, could alleviate some of the burdens faced by students in the online learning setting.

Our findings underscore the need for policymakers and educational institutions to prioritize student satisfaction in online learning environments. This can be achieved by ensuring the availability and accessibility of the necessary tools and updated infrastructure for smooth course delivery. Additionally, students should have confidence in completing assignments and tests, and access to emotional support when needed. Effective communication channels between students and faculty, even in the absence of in-person interactions, are crucial. Faculty members should receive mandatory training or workshops to acquire the necessary skills for delivering effective online lectures.

Furthermore, it is essential to conduct long-term research to understand the effects of online learning on students’ future endeavors and careers, particularly for those who require hands-on learning experiences. This will enable educators and policymakers to make informed decisions regarding designing and implementing online learning environments, ensuring that they align with students’ long-term goals and career aspirations.

Future research could delve deeper into the reasons behind observed gender preferences for online and face-to-face learning. For example, does the level of digital literacy play a role, or do social dynamics within the online classroom environment impact student preferences? Our study did not explore these potential factors, which could offer valuable insights. Additionally, since our study was confined to Zayed University, further research involving diverse institutions across the UAE or even globally could provide a more holistic understanding of online learning during pandemic situations.

In conclusion, our study highlights the significance of student satisfaction for successful online learning. By addressing the challenges and implementing strategies to enhance student satisfaction, educational institutions can create a conducive environment that promotes effective online learning and supports students in achieving their educational goals.

## Data availability statement

The original contributions presented in the study are included in the article/supplementary material, further inquiries can be directed to the first author.

## Ethics statement

This study was approved by the Research Ethics Committee (REC), Office of Research at Zayed University, Ethics Application Protocol Code ZU21_169_F, and the Ministry of Health and Prevention Research Ethics Committee, Ethics Application Protocol Code MOHAP/DXB-REC/ JJF/No. 05/2022. The participants provided their written informed consent to participate in this study. Written informed consent was obtained from the individual(s) for the publication of any identifiable data included in this article.

## Author contributions

All authors listed have made a substantial, direct, and intellectual contribution to the work and approved it for publication.

## Funding

This study has been funded by Zayed University, Office of Research, Emirati Research Grant Award, Activity Code R22057.

## Conflict of interest

The authors declare that the research was conducted in the absence of any commercial or financial relationships that could be construed as a potential conflict of interest.

## Publisher’s note

All claims expressed in this article are solely those of the authors and do not necessarily represent those of their affiliated organizations, or those of the publisher, the editors and the reviewers. Any product that may be evaluated in this article, or claim that may be made by its manufacturer, is not guaranteed or endorsed by the publisher.
